# Transportation considerations in underserved patient populations receiving multidisciplinary head and neck cancer care

**DOI:** 10.3934/publichealth.2024058

**Published:** 2024-12-03

**Authors:** Luke Stanisce, Donald H Solomon, Liam O'Neill, Nadir Ahmad, Brian Swendseid, Gregory J Kubicek, Yekaterina Koshkareva

**Affiliations:** 1 Division of Otolaryngology – Head & Neck Surgery, Cooper University Health Care, Camden, NJ, USA; 2 Cooper Medical School of Rowan University, Camden, NJ, USA; 3 Head & Neck Cancer Center, MD Anderson Cancer Center at Cooper, Camden, NJ, USA; 4 Department of Radiation Oncology, University of Miami Health Systems, Miami, FL, USA

**Keywords:** social determinants, transportation, underserved, multidisciplinary, head and neck cancer

## Abstract

**Background:**

Underinsured patients with advanced head and neck cancer experience worse outcomes compared to their well-insured peers.

**Methods:**

Retrospective logistic regression analysis testing associations between demographic, geospatial, transportation, disease, and treatment factors in 50 government insured or uninsured patients receiving curative-intent, multidisciplinary cancer care.

**Results:**

Forty percent of patients missed at least one treatment or surveillance appointment within the first year. Thirty-two percent reported using public transportation; 42% relied on caregivers. Patients who used public transportation were 3.3 and 4.6 times more likely to miss treatment (p = 0.001) and surveillance (p = 0.014) visits, respectively. The median one-way travel duration for such routes was 52 minutes (range: 16–232 minutes) and included 0.7 miles of walking. Physical distance to care was not associated with transportation type, missed appointments, or disease recurrence.

**Conclusions:**

Underserved, underinsured patient populations face significant logistical challenges with transportation, which may be mitigated by alternative models of care delivery, such as multidisciplinary clinics.

## 1. Introduction

A multitude of contemporary investigations have demonstrated associations between various socioeconomic factors and worse outcomes of locally advanced head and neck cancer. Underinsured patients and ethnic minorities exhibit significantly higher mortality rates, while limited access to care (further distances to hospitals and rural residences) is linked to inferior outcomes [1]–[6]. Delays in treatment initiation, in part driven by missed appointments or decreased patient health literacy, preferentially affects underserved populations [4]. As the regionalization of care continues to evolve and patients must travel longer distances for complex oncological treatment, these trends and discrepancies may intensify [7],[8].

With an emphasis placed on survivorship in head and neck cancer care, the financial and psychological burdens associated with treatment and surveillance may go overlooked. Both direct and indirect costs of complex oncological care can be immense, including transportation, lodging, and personal and caregiver time off work [9]. Unique to head and neck cancer, disease and treatment may result in unconcealable deformities and dysfunction. It stands to reason that underserved populations may be disproportionately impacted from both economic and emotional perspectives [10],[11].

Located in an urban city where an estimated 40% of the population lives in poverty, our academic tertiary-care institution serves a large demographic of socioeconomically challenged patients. To better serve this unique population, we aimed to identify barriers of care amongst patients with advanced head and neck cancer. This report quantifies underrecognized challenges with transportation faced by such patients. Concurrently, we share a personal correspondence received from a patient in our practice to highlight the rigors of public transportation during one's treatment phase. An enhanced understanding of these social determinants will provide practitioners with insight to address the current discrepancies in head and neck cancer care.

## 2. Materials and methods

Following approval from our Institutional Review Board (#19–063EX), we reviewed the electronic records of in-state patients from our institution's multidisciplinary Head and Neck Tumor Board Registry database whose insurance coverage was either “Medicaid,” “Medicare,” or “Uninsured” from January 2015 to December 2017. Patients with either supplemental insurance or ambiguous coverage were excluded in order to best capture the underserved population. Patients whose treatment recommendation was not curative in nature were excluded. Patients who received adjunct treatment at an outside institution were excluded. From this cohort, 50 patients were randomly selected using a random number generating software. Retrospectively reviewing physician, nurse navigator, social work, and demographic documentation, the following information was extracted and recorded into a single dataset: age; gender; race/ethnicity; marital status (married, single, divorced, or widowed); smoking and alcohol usage (current, previous, never); primary tumor location and type; TNM classification; date and location of diagnosis (our institution versus outside institution); treatment modalities; incidence of treatment toxicity; home address; location of scheduled treatment and clinic visits; transportation utilized to get to and from appointments (when information was available); the incidence of missed treatment or surgical, medical oncology, and radiation oncology surveillance appointments within one-year post-diagnosis; the reason given for a missed appointment (when available); the incidence and date of recurrence; and the date of the last known follow-up.

All data collection and analysis were completed using our institution's secure institutional network. The number of missed treatment visits was inconsistently captured in the electronic medical record and was extrapolated from physician and nurse navigator notes in select cases. Thus, the incidence of missed treatment or surveillance appointments were calculated as dichotomous endpoints. The method of transportation utilized was not available for every appointment. To reduce heterogeneity for statistical analysis purposes, the following categories were used: public, caregiver, and professional (i.e., medical transportation, taxi service, ride sharing applications). Google Maps software (Google LLC. Mountain View, CA, USA) was used to determine the physical distance between patients' home addresses and our institution. Multiple transportation routes were calculated for each scenario: via public transportation using Google Maps software (the route with the shortest travel time), and via digital ride-hailing options using the Lyft and Uber mobile applications (Lyft, Inc. San Francisco, CA, USA; Uber Technologies, Inc. San Francisco, CA, USA). The cost associated for each option was calculated and recorded, excluding routine costs such as vehicle maintenance, tolls, and gasoline. All routes were calculated under the assumption of optimal travel and traffic conditions, and did not account for variability based on demand.

Descriptive statistics were calculated for the study cohort and baseline associations between variables were calculated using Pearson Chi-square and Man-Whitney U testing. Univariate logistic regression analysis was used to assess the associations between continuous or categorical variables and endpoints of interest. The primary endpoints of interest were the incidence of missed treatment and surveillance visits. Secondary endpoints were the incidence of disease recurrence. Odds ratio (OR) estimates with 95% confidence intervals (CI) were calculated via Wald Chi-square and Fishers exact testing. Box-Tidwell procedure was used to confirm the linear relationship between continuous variables (age, distance) and the logit transformation of the binary outcomes. Multivariate factorial binominal logistic regression analysis was executed, but found to be statistically insignificant likely restricted by the sample size. Kaplan-Meier analysis was utilized to estimate recurrence-free survival, and log-rank test was used to assess differences by groups. Patients were censored accordingly at the date of last assessment, loss to follow up, or death. Cox proportional regression methodology was used to analyze recurrence-free survival by various patient, disease, and treatment factors. P < 0.05 using 95% confidence intervals was considered statistically significant. All statistical analysis was conducted using SPSS (IBM SPSS Statistics Software, Version 27.0. Armonk, NY, USA).

## 3. Results

The median age of the study cohort was 61 years old (range, 46–92). Thirty-one (62%) patients were male. Thirty-three (66%) patients identified as “White/Non-Hispanic,” 13 (26%) as “African American,” eight (16%) as “Latino-Hispanic,” three (6%) as “Asian,” and three as “Other/Deferred.” Fourteen (28%) patients were married, 19 (38%) were single or never married, 11 (22%) were divorced, and six (12%) were widowed. Twenty (40%) patients identified as current everyday smokers at the time of treatment initiation, 19 (38%) as previous smokers, and 11 (22%) as never smokers. Fifteen (30%) patients reported current daily alcohol use, 13 (26%) as previous daily alcohol consumers, and 22 (44%) as never-drinkers.

Laryngeal cancer was the most common primary site (n = 24), followed by the oral cavity (n = 18), oropharynx (n = 7), and sinonasal region (n = 1). Squamous cell carcinoma attributed for 47 (94%) of the neoplasms. Two patients had adenoid cystic carcinomas, and one had adenocarcinoma. Based on AJCC 7th edition, 36% of patients had T4 classified lesions upon presentation (n = 18), and 44% of patients had N2 disease (n = 22). Forty-two (84%) patients underwent surgical extirpative procedures. Forty-eight (96%) patients underwent radiation treatment, 38 (76%) of which were adjuvant therapy. Chemotherapy was included in twenty treatment regimens (14 adjuvant concurrent chemoradiation, six primary chemoradiation). Eleven (22%) patients experienced treatment-related toxicity, all of which were CTCAE Grade 2 or less. Patient, disease, and treatment characteristics are summarized in Table 1.

**Figure 1.**
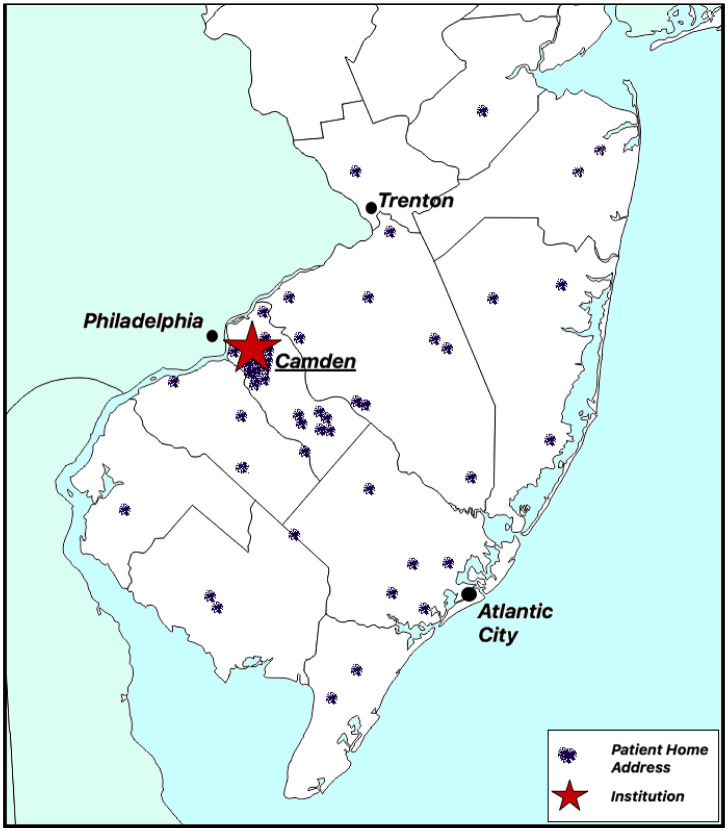
Map of New Jersey and the area surrounding our institution visually depicting the distance from each patient's home address.

The median distance from home address to our institution was 19.9 miles (range: 0.8–72.8 miles). Nineteen (38%) patients lived within the same state county as our institution, 19 lived within bordering counties, and 12 (24%) were from nonbordering counties. Sixty-eight percent of cases (n = 34) were diagnosed at our institution. Visual representation of the geographical region in relation to the patient cohort are illustrated in Figure 1.

Dependability on caregiver transportation was reported by 42% (n = 21) during the first year of treatment. Thirty-two percent of patients (n = 16) reported using public transportation. Public transportation options were not available for eight patients. Thirty-five patients (70%) utilized a method of professional transportation (i.e., medical transportation) during the first year. The use of a personal ride-sharing application was noted in four (8%) of the patient's records.

For the 16 patients who used public transportation, the median shortest travel time was 52 minutes (range: 16–232 minutes). Eight (50%) of these routes did not require a transfer, four (25%) routes required at least one transfer, three (19%) required two transfers, and one required three transfers. The public transportation routes required a median of 0.7 miles of walking (range: 0.27–1.4), estimated to take a median of 16 minutes (range: 4–30 minutes).

Patients who were divorced were 5.8 times more likely to utilize public transportation compared to those who were not (p = 0.016, OR 5.83, CI 1.4–24.5). Widowed patients were 1.4 times more likely to utilize family transportation compared to non-widowed patients (p = 0.003, OR 1.40, CI 1.1–1.8). There were no differences between the type of transportation utilized and patient race/ethnicity, age, alcohol and tobacco use, and tumor staging. There was no association between the distance (continuous and categorical) from our institution and the type of transportation used.

A total of 20 (40%) patients missed either or both a surveillance or treatment appointment in this first year. Nineteen patients (38%) missed surveillance appointments, and 12 (24%) missed treatment visits. Transportation was listed as a reason for a missed appointment in 45% of the patient's charts (n = 9). Family tragedy was captured as a reason for a missed appointment in one case. Information about reason for missed appointments was not available for the remainder of cases.

Those patients who used public transportation were 3.3 and 4.6 times more likely to miss treatment and surveillance visits, respectively, compared to those who did not (p = 0.001, CI 2.8–8.7; p = 0.014, CI 1.3–16.4). The use of caregiver and professional transportation were not significantly associated with missed appointments. Current tobacco usage was associated with increased odds of missed treatment (p = 0.035, OR 4.33, CI 1.1–17.3) and surveillance appointments (p = 0.043, OR 3.36, CI 1.02–11.1). Inversely, those who never used tobacco were 1.4 and 8.3 times less likely to miss a treatment or surveillance visit compared to those who used tobacco (p = 0.032, OR 0.71, CI 0.58–0.87; p = 0.025, OR 0.12, CI 0.01–0.93, respectively). Similar trends were seen with current (OR 3.22) and never (OR 0.49) alcohol usage and missed treatment visits. Neither distance or diagnosis at an outside institution was associated with the incidence of missed visits. Gender, race/ethnicity, age, marital status, primary site, staging, and treatment toxicity did not predict for missed appointments. Complete univariate analysis is summarized in Table 2.

**Table 1.** Patient, disease, and treatment characteristics of the study cohort.

Characteristics	No. of patients (%)
Gender-Male	31 (62)
Age-median (years), range	61 (46–92)
Race/Ethnicity	
*White*	33 (66)
*African American*	13 (26)
*Latino/Hispanic*	8 (16)
*Asian*	3 (6)
*Other*	3 (6)
Marital Status	
*Married*	14 (28)
*Single*	19 (38)
*Divorced*	11 (22)
*Widowed*	6 (12)
Tobacco	
*Current*	20 (40)
*Previous*	19 (38)
*Never*	11 (22)
Alcohol	
*Current*	15 (30)
*Previous*	13 (26)
*Never*	22 (44)
Larynx	24 (48)
Oral	18 (36)
Oropharynx	7 (14)
Sinonasal	1 (2)
*T* Stage	
T1	10 (20)
T2	13 (26)
T3	9 (18)
T4	18 (36)
*N* Stage	
N0	19 (38)
N1	8 (16)
N2	22 (44)
N3	1 (2)
Surgery	42 (84)
Radiation	48 (96)
Chemotherapy	20 (40)
Toxicity	11 (22)

**Table 2.** Results of univariate analysis testing the association of patient, disease, geospatial, and transportation factors with missed treatment and surveillance appointments. Statistically significant predictors are highlighted in bold.

Factors	Missed treatment appointment(s)			Missed surveillance appointment(s)		
	p	OR	95% CI	p	OR	95% CI
Gender, Male	0.081	4.05	0.78–21.0	0.15	2.31	0.66–7.9
Age	0.86	1.01	0.95–1.1	0.31	0.97	0.91–1.03
Distance	0.22	1.02	0.98–1.1	0.22	2.18	0.63–7.5
Race/Ethnicity*	0.83			0.27		
Marital Status*	0.19			0.48		
Tobacco						
*Current*	0.035	4.33	1.1–17.3	0.43	3.36	1.02–11.1
*Previous*	0.49	0.77	0.2–3.0	0.89	0.92	0.28–3.1
*Never*	0.032	0.71	0.58–0.87	0.025	0.12	0.01–0.93
Alcohol						
*Current*	0.083	3.22	0.83–12.5	0.14	2.49	0.72–8.6
*Previous*	0.149	2.68	0.67–10.7	0.17	2.43	0.67–8.8
*Never*	0.006	0.49	0.01–0.63	0.011	0.19	0.05–0.72
County*	0.49			0.74		
Public Transportation	0.001	3.26	2.8–8.7	0.014	4.63	1.3–16.4
Family Transportation	0.49	0.62	0.16–2.4	0.56	0.71	0.22–2.3
Professional Transportation	0.30	2.60	0.49–13.7	0.12	3.37	0.81–14.1
Diagnosed at CUH	0.91	0.92	0.23–3.7	0.96		
Anatomic Location Primary*	0.39			0.59		
Toxicity	0.24	2.24	0.52–9.5	0.41	1.49	0.38–5.7
T Classification*	0.35			0.088		
N Classification*	0.63			0.24		

Note: OR = Odds ratio; CI = Confidence interval; *ORs and 95% CIs values not shown for each categorical predictor for the nominal variables that did not reach significance.

The median length of follow up was 992 days (range: 79–2284 days). Sixteen (32%) patients experienced locoregional recurrence or treatment failure. The median time to recurrence was 403 days (range: 25–1607). Both missed treatment and missed surveillance appointments within the first year were associated with the incidence of recurrence (p = 0.003, OR 1.40, CI 1.1–1.8; p = 0.003, OR 1.40, CI 1.1–1.8, respectively). All other factors, including TNM classification and distance, were not statistically associated with recurrence.

## 4. Discussion

The findings in this single-center cohort demonstrate that underserved patients undergoing multidisciplinary head and neck cancer treatment face a variety of obstacles related to transportation. Moreover, these results suggest that transportation barriers may hold significant clinical implications. By identifying high-risk demographic and geospatial predictors, this review aims to improve our current understanding of limitations to ideal oncologic care and clarify relationships between travel, access, and outcomes.

Prior literature assessing national cancer databases has associated increased travel distances with improved outcomes in head and neck cancer care [12]–[14]. Initially thought to reflect patients traveling further to seek higher quality of care, Massa et al. alternatively proposed that travel distance is a surrogate measure of patent factors and socioeconomic resources. Their group hypothesized that this survival benefit is likely the effect of unmeasured confounding, as patients with the resources to voluntarily travel further may have other protective advantages [13]. Exclusively selecting for a disenfranchised cohort, we observed that 40% of such patients missed appointments in the first year of treatment regardless of travel distance. Rather than distance itself, challenges with travel like dependence on public transportation were linked to missed appointments and worse outcomes. Additional investigations at the patient-specific level, like that of our cohort, are needed to better clarify these relationships.

This report highlights the necessity to further allocate institutional resources to assistance programs focusing on ancillary patient needs. Providers are tasked with imparting effective transportation services to vulnerable patients undergoing complex oncologic care. Similar to many tertiary care institutions, our organization has team members dedicated to facilitating care, providing available resources, and navigating such issues. Medicaid has developed state-specific programs to provide eligible beneficiaries with non-emergency medical transportation [15]. Nonetheless, these factors may go overlooked as evident by our own review, let alone by institutions without dedicated resources or experience caring for socioeconomically challenged populations.

In complement to traditional medical transportation, the digital transportation network companies Lyft and Uber offer an alternative option. However, the cost associated with such services can be immense and not feasible for underserved patients. Only four patients from our cohort reported their use. To demonstrate the financial burden of these services, the median one-way cost of using a private, digital ride-hailing method of transportation was $44.53 (range: $7.80–$179.67). The median one-way cost of using a shared, digital ride-hailing option was $37.36 (range: $6.81–$152.75). To counter such costs, both companies have backed initiatives to fulfill third-party, health care-related ride requests. These services are named Concierge and Circulation, respectively [16],[17]. However, their applicability to underprivileged populations and certain regions may be limited by smartphone accessibility and driver penetration to rural areas [18].

As efforts to reform the delivery of care proceed, it is essential that we utilize patients as a valuable resource and barometer to measure change. The following passage was written by a patient in a personal correspondence with our team when discussing the transportation options available:

*My “Highway to Healing” began in February of 2017 when I was diagnosed with advanced tongue cancer. My treatment consisted of a triad of medical, surgical, and radiation specialists who carried me through seven weeks and 35 daily visits for chemotherapy and radiation treatments. In the proceeding twenty months, I would take nearly 50, 2-hour bus rides from Atlantic City, NJ to Camden, NJ, then back home. Each trip was fraught with frustration, anxiety, and anticipation. What news would I receive? How would I feel after treatment? What would the person next to me think about my trach? Or portable suction? This journey and these emotions eventually became routine through diagnosis, treatment, and after*.

*I am grateful to all for the exceptional care I have received from my teams and their staff. However, my passage to recovery and remission may have been considerably easier, faster, and more convenient without the physical, psychological, and economic obstacles faced... Simplifying the process would have significantly lessened many of the hardships I endured as it actually took place*.

When focusing on treatment outcomes and survival, it is easy to overlook the subtle nuances mentioned above; the prolonged uncertainty of receiving unsettling news and having to cope with bad news in a public setting. These elements may be intensified and specific to head and neck cancer due to the burden of specialized equipment like suctioning devices or the cosmetic deformities resulting from disease or treatment. These logistical and psychological considerations faced by head and neck cancer survivors are unique and may be more prominent in those of underserved populations relying on public transportation. When asked how to make visits easier and less stressful, Massa et al. found that head and neck cancer patients desire both the reduction of transportation difficulties and frequency of clinic visits [19].

As proposed by Graboyes et al., the model in which we delivery care should serve as a modifiable target for changing head and neck cancer outcomes [12],[20]. Within the last decade, the multidisciplinary clinic (MDC) model of care has been introduced at select institutions, in which surgical, medical, and radiation oncology appointments are streamlined into a single day. Initial studies analyzing head and neck cancer MDCs have reported improved access to multimodal therapy [21]–[24]. However, no studies no date have demonstrated their applicability in underserved communities, although the theoretical benefits of this care model seem ideal.

Liao et al. demonstrated that treatment delays and worse outcomes in urban patients are most commonly due to missed appointments and treatment refusal [4]. Our review demonstrated that missed appointments was associated with disease recurrence. By limiting the number of appointments, and reducing the burden of transportation, the MDC model may lessen missed appointments in underserved patients. In a similar vein, lower socioeconomic status is linked to a worse level of medical literacy [25]. In the MDC model of care, perspectives from various ancillary providers including speech language pathology, audiology, and nutritionists may provide patients with additional information and help to address the underlying motives for treatment refusal.

Our cohort may reflect a marginalized population, potentially influenced by the effects of the marginalization-related diminished returns theory [26],[27]. This concept explores the reduced efficacies of various interventions amongst marginalized people, due to broader societal processes such as racism and stratification. Despite improving factors such as access, worse than expected outcomes may still be observed amongst some of our subgroups.

There are several limitations to this report and discussion. This was a single center analysis restricted by the retrospective nature of data collection and small sample size. Future multi-center prospective studies are warranted to more consistently and quantitatively capture patient perspectives. Inherent to the statistical analyses, certain associations may not maintain significance with more robust cohorts. Moreover, the variables assessed are often not mutually exclusive. There is likely a high level of multicollinearity amongst various socioeconomic factors and critical interactions between each predictor. To correct for this, we attempted to conduct multivariate factorial analysis; however, the results were limited by the sample size. Larger population-based analysis is required to uncover the true effect and associations these factors hold. Numerous factors, including education, income, and psychological outcomes, were not included due to the retrospective nature of data collection. These aspects of patient-centered care serve as targets of interest for future studies. Finally, prospective studies applying the MDC model in underserved populations need to be conducted. Since health literacy and socioeconomic status are closely related, foreseeable limitations exist (i.e., patient understanding or retention may decrease as more information is given in a single-day multi-provider visit).

## 5. Conclusions

Head and neck cancer patients have to balance numerous surgical, medical, and radiation oncology appointments over the course of their treatment, which can be practically and financially challenging. The analysis presented herein highlights the underrecognized magnitude of transportation challenges faced by patients of an underserved population and the clinical implications they hold. Practitioners should continue to explore changes to policy and delivery of care in an effort to best advocate for their patients.

## Use of AI tools declaration

The authors declare they have not used Artificial Intelligence (AI) tools in the creation of this article.
